# Public knowledge of people visiting Imam Reza hospital regarding stroke symptoms and risk factors

**DOI:** 10.1186/s12873-019-0250-5

**Published:** 2019-06-28

**Authors:** Elyar Sadeghi-Hokmabadi, Samad Shams Vahdati, Reza Rikhtegar, Khazar Ghasempour, Aysa Rezabakhsh

**Affiliations:** 1 Neurosciences Research Center (NSRC), Tabriz University of Medical Sciences, Tabriz, Iran; 2 Emergency Research Team, department, Imam Reza Hospital, Tabriz University of Medical Sciences, 5166/15731, Golghasht street, Tabriz, Iran; 3 Medical student, School of Medicine, Tabriz University of Medical Sciences, Tabriz, Iran; 4 Aging Research Institute, School of Medicine, Tabriz University of Medical Sciences, Tabriz, Iran

**Keywords:** Stroke risk factors, Public knowledge, Warning symptoms

## Abstract

**Background:**

Early recognition of stroke symptoms results in a lower time period after stroke onset to treatment with a better outcome. This depends on the awareness of patients, family members, and the general public.

**Objective:**

The aim of this study was to evaluate public awareness about stroke risk factors, warning symptoms, and treatments.

**Methods:**

This cross-sectional study was conducted as a hospital-based survey on 2712 people who visited clinics or emergency department of Imam Reza hospital for any reason, from March 2015 to February 2016. All subjects were interviewed face-to-face by four trained physicians and a structured, pre-tested questionnaire was filled.

**Results:**

The mean age of participants was 41.0 ± 12.1 years old. Considering Cincinnati prehospital stroke scale (CPSS) as the main diagnostic system, the percentage of participants that mentioned face asymmetry, speech disturbances, and arm paralysis as a symptom of stroke was 7, 1.5, and 7.9%, respectively. Meanwhile, 71.2% of participants could not mention any of the stroke symptoms. Among participants, 20.2% did not know any of stroke risk factors although 35.1, 27.8, and 17.3% could name one, two and three or more risk factors, respectively. Among participants, only 1.1% were aware of thrombolytic therapy (t-PA) as a first-line drug for stroke treatment.

**Conclusion:**

In this study, public knowledge regarding stroke symptoms, risk factors, and therapy approaches was low. Taken together, public education is necessary to reduce the time for recognition of stroke symptoms and subsequently prompt and proper proceeding seems to be necessary for the community.

## Background

Stroke is the main reason of disability and the second leading cause of death globally [1]. Annually, approximately 17 million people suffer a stroke which more than 6 million dies and a considerable number remain to disable [1]. Iran is a developing country with a stroke prevalence of 23–139 per 100,000 populations. Incidence of stroke in Iran is considerably higher than most Western countries with a stroke occurring approximately one decade earlier in life. Stroke per se has a great socio-economic burden for families and the whole society [2, 3]. Recent data have been shown that one of the main barriers for receiving thrombolytic therapy is associated with a delay in the arrival of Iranian patients at the hospital [4–6]. Early recognition of stroke symptoms and early arrival at a hospital will provide greater opportunity for an effective stroke management [7]. Public awareness regarding stroke symptoms can be critical and it could decrease time period after stroke onset to hospital presentation [8]. Moreover, population-based studies have shown that there is little knowledge about stroke symptoms and risk factors in different countries [9, 10]. In Iran, data about stroke are low and mainly limited to studies carried out in Shiraz and Bushehr, South of Iran. The results showed that knowledge of stroke in the general population was acceptable [11]. In order to increase general awareness of stroke, public education is required. It seems that effective educational planning relies on an accurate assessment of the baseline knowledge of the population. In this study, we aimed to evaluate the awareness level of the people referring to Tabriz Imam-Reza hospital, on the details of stroke symptoms, its risk factors, and treatments.

## Methods

### Study design

A cross-sectional hospital-based survey was conducted among 2712 subjects who visited Imam-Reza outpatient clinic and emergency department for any reason from March 2015 to February 2016. Imam-Reza hospital is the tertiary university hospital where is the main referral center in the Tabriz, northwest of Iran. All subjects were interviewed face-to-face by four trained physicians and a structured, pre-tested questionnaire was filled by these physicians. The interviewers interrupted the interview only to clarify a question if required, but they did not reveal any information about the questions. Eligible candidates were individuals over 18 years old, with the ability to communicate in common regional language (Turkish or Persian) and were inclined to participate in the study.

### Questionnaire

A literature review of previous studies regarding stroke knowledge and risk factors was done to identify potential items for the questionnaire. Then, these items were modified by the authors to cope with local sociocultural issues. The questionnaire was pretested using a sample of 30 people. Changes were made in the questionnaire to various terms that are used for “stroke” in the local language (Turkish). The questionnaire consisted of 5 sections: (1) demographic information which concerned respondent’s age, sex, education, race, occupation, income and place of residence (urban or rural), (2) stroke warning signs, (3) stroke risk factors, (4) stroke treatment, in this section two questions were asked from responders (a) “ if someone appeared to have a stroke, what would you do first?” (b) “What is an appropriate treatment option for stroke?” and (5) at the last section the source of information they obtained about stroke was asked.

### Statistical analysis

After filling out the questionnaires, the gathered data were analyzed by SPSS ver.17 software package (SPSS Inc., Chicago, IL, USA). For evaluating the descriptive statistics, data were represented as mean ± standard deviation (SD) and frequency percent. In order to evaluate the possible relations between various factors Pearson regression test was used.

## Results

### General characteristics of the respondents

According to our data, the mean age for patients was 41.02 ± 12.18 years old. Within the subjects, 1576 (58.1%) were male and female accounted for 1136 (41.9%). Only 36.1% of the referring patients had academic educations (i.e. bachelor’s and master’s or Ph.D. degree) and the rest (62.9%) were either illiterate or had primitive literacy. There was a significant inverse relationship between patients’ age and level of education and with aging the literacy rate was lower (*r* = 0.056). Also, female patients had more academic degrees compared to males. Most of the patients were private employees (36%) (i.e. working in non-governmental settings like constructions) and housewives (31%), while others were as follows; 7% students or retired employees, 4% farmers, 3% employees and technicians, and the rest (2% each) teachers or nurses. Among participants, 1762 (65%) were residents of big cities, 605 (22.3%) were belonged to small cities and the rest (12.6%) were inhabitants of rural areas. Of note, most of the illiterate patients with lower literacy rate belonged to rural areas. Economically, of patients were classified into three main groups; low income (74.7%), median income (24.7%) and high income (0.6%).

### Knowledge of stroke warning signs

Subjects were aware of 1.1 ± 0.6% of the 11 mentioned symptoms in the questionnaire with a minimum of 0 and maximum of 6 symptoms. The most common warning signs of stroke by participants were “Sudden numbness or tingling sensation” identified by 412 respondents (15.2%), and “Sudden paralysis of one side of the body” identified by 215 respondents (7.9%). The details are outlined in Table 1. Considering CPSS (Cincinnati prehospital stroke scale), the number of participants that mentioned face asymmetry, speech disturbances, and arm paralysis as a symptom of stroke was 191 (7%), 117 (4.3%) and 215 (7.9%), respectively. Only 19.2% of participants mentioned at least one of Cincinnati items as a stroke warning sign, while 1931 (71.2%) could not mention any of stroke symptoms.

**Table 1:** Knowledge of stroke warning signs

Signs	Frequency	Percent
Sudden numbness or tingling	412	15.2%
Sudden drop	316	11.7%
Loss of consciousness	276	10.2%
Sudden palsy	215	7.9%
Facial palsy	191	7%
Speech disorder	117	4.3%
Sudden headache	79	2.9%
Acute vertigo	53	2%
Sudden blindness	23	0.8%
Acute cognition disorder	17	0.6%
Acute visual error	15	0.6%
Others	42	1.5%
No knowledge	1931	71.2%

### Knowledge of stroke risk factors

Unfortunately, participants were only aware of 1.4 ± 1.1% of the 12 mentioned risk factors in the questionnaire with a minimum of 0 and maximum of 8 risk factors. The details are illustrated in Table 2. Among participants, 549 (20.2%) did not know any of stroke risk factors although 35.1% (925) could name 1 risk factor correctly, 27.8% (754) named 2 risk factors, and 17.3% (469) named 3 or more risk factors.

**Table 2:** Knowledge about stroke factors

Factors	Frequency	Percent
Stress	1787	65.9%
Hypertension	1040	38.3%
Diabetes Mellitus	438	16.2%
Obesity	187	6.7%
High blood lipid	177	6.5%
Smoking	157	5.8%
Cardiovascular disease	88	3.2%
Hereditary	41	1.5%
Old age	38	1.4%
Alcohol	29	1.1%
Lack of physical activity	29	1.1%
Other factors	44	1.6%
No information	549	20.2%

### Predicting factors for awareness of stroke symptoms and risk factors

Because of the education level increased in this study, patient’s knowledge about the disease and related risk factors increased significantly (*p* < 0.01). Moreover, patients awareness of stroke symptoms and risk factors were increasing while the region of the living moved from bigger cities to smaller ones and finally villages (*p* < 0.01). Though female subjects were more familiar with symptoms and risk factors, there was no statistically significant difference between genders knowledge (*p* = 0.1). On the other hand, there was a significant relationship between age and awareness of the symptoms or risk factors (*p* < 0.01), while the reported correlation was too low (*r* = 0.056 and *r* = 0.067, respectively).

### Respondent’s reaction to stroke symptoms

When participants were asked what they would do first in case of facing somebody with acute symptoms of stroke, 59% stated that they would call the EMS (Emergency medical services). The detailed respondent’s reaction to stroke Symptoms is shown in Table 3.

**Table 3:** Reaction to stroke

Reactions	Frequency	percent
Call EMS	1601	59%
Transfer to hospital	828	30.5%
Call family	175	6.5%
Transfer to clinic	84	3.15
Transfer to Physician office	6	0.2%
Call physician	3	0.1%
Others	15	0.6%

### Knowledge of stroke treatment

Among all, 2537 of subjects (93.5%) had no idea about treatment, 122 subjects (4.5%) mentioned aspirin taking and 23 subjects (0.8%) mentioned surgical approach as the treatment of stroke. Only 30 subjects (1.1%) were aware of thrombolytic therapy such as t-PA (tissue plasminogen activator) as a selective treatment for stroke.

### Sources of information

When the subjects were asked regarding their source of knowledge about stroke, most of them mentioned relatives and friends as a source of acquaintance with this disease. Detailed information in this regard is brought in Table 4. Comparing subjects of rural areas with urban regions showed that residents of both rural and urban areas tend to have most of their knowledge through relatives and then TV or public media, which was 83.1 and 8.2% of subjects for rural residents and 57.6 and 27.1%, within the urban areas, respectively.

**Table 4:** Sources of information

Sources	Frequency	Percent
Family and relatives	1033	38.1%
Television	660	24.3%
Previous stroke	453	16.7%
Physicians	313	11.5%
Newspaper and magazines	260	9.6%
Medical books	105	3.9%
Medical staffs	53	2%
Radio	21	0.8%
Others	30	1.1

## Discussion

The epidemiologic study revealed that stroke has been reported to be the second cause of death in Iran after cardiovascular diseases [2]. Demographic results of this study showed the low socioeconomic condition of the participants, including low academic degrees (36%) and low income (74.7%). Based on the patient’s knowledge assessment related to the stroke symptoms and risk factors, 71.2 and 20.2% of subjects declared no knowledge, respectively. The similar results were reported by Jones et al. in 2010 about the knowledge of subjects about stroke [12]. In line with our results, most of the studies showed narrow knowledge about stroke within older people and patients with lower education levels. Ultimately, Jones et al. demonstrated that the patient’s knowledge of different studies regarding stroke was poor. In a study performed by Wiszniewska and colleagues, the knowledge of non-stroke patients was evaluated regarding the risk factors and symptoms [13]. Among 481 patients, 91.1% of them named one risk factor (hypertension) for stroke, while diabetes and cardiac arrhythmia were not well-known factors. On the other hand, 25% of them knew nothing about stroke symptoms. The symptoms that patients were aware of them mostly included the disturbances of consciousness, numbness, and dizziness, whereas, in this study sudden numbness, sudden fall and loss of consciousness were the leading well-known symptoms. It is noteworthy that most patients from rural areas were unaware of stroke signs and symptoms. In a study conducted by Aboutalebi et al. 83% of patients named at least a symptom and 95% named at least one risk factor of stroke in Bushehr city were showed the high awareness of patients in comparison to our findings [14]. Another study by Hatzitolios revealed that 24.1% of the subjects, responding to open-ended unaided questions, were unaware of stroke symptoms, and the most well-known symptom was arm or leg weakness [9]. Among participants, 32.7% mentioned one risk factor, while 32.7% were totally unaware of any of the risk factors. Unlike our study, older subjects knew more about stroke in case of facing stroke and mentioned that they would take the patient to the hospital by their own private car. Additionally, the knowledge of stroke risk factors and warning signs were evaluated according to asking quota-based questions by telephone calls and 71% of subjects listed two or more risk factors for stroke (54.7% smoking and 41.2% being over-weight), which was higher than our results [15]. Monaliza et al., also assessed the visitors of internal and surgery wards, for their knowledge regarding stroke symptoms and find out 96.15% of participants were aware of one of the well-known symptoms such as sudden numbness or weakness of the face, arm, or leg especially one side of the body [10]. On the other hand, 52.87% of them knew about the risk factors such as hypertension (58.45%) and alcohol consumption (48.82%). Similar to our study, participants with younger ages, higher academic degree, and income tend to know more about stroke warning signs and symptoms. In contrast, Moreira et al. declared that older ages more awareness about stroke disease [16]. In the systematic review conducted by Stroeble et al., they showed that the knowledge of female subjects was higher than male subjects which were compatible with our result [17]. In line with our findings, Truelsen et al. found similar results about higher awareness among females in compare with male patients regarding both symptoms and risk factors of the disease [18]. Another study demonstrated that the most common risk factors were hypertension (88.8%) and smoking (87.8%) and the most common warning signs were abdominal and chest pain [11]. Similar to our study, most of the participants chose to take the patient to the emergency room or call EMS. They concluded that people in Shiraz province, need to be educated regarding the stroke risk factors, symptoms, and the first action in case of the stroke. It is necessary to mention that our study conducted in a single place with rather a small size of samples; therefore we could not generalize our findings to other population. Additionally, racial and ethnic differences have not been considered.

### Strength/limitation

Imam Reza and Razi hospitals are two referral and high volume medical centers with patient admission from different urban/rural regions of north-west of Iran. Therefore, our results regarding to public knowledge could be more reliable and authentic than other local medical centers. Moreover, up to now the evidence based educational practice is not available in North-west of Iran especially in rural regions about stroke symptoms. So our data extracted from this study could be more valuable to raise awareness level of people through the most appropriate way possible. However, the racial and ethnic differences could not be considered in our study. Hence, the further study is required to evaluate the probable impacts of the racial and ethnic differences in public knowledge about stroke symptoms.

## Conclusion

Participants with higher education, income level, and younger age tend to know more about stroke symptoms and related risk factors. Results of this study showed lower awareness of subjects in Tabriz in comparison to most of the urban areas worldwide. The lower level of knowledge might result in reduced control of lifestyle and subsequently caused the increased level of risk factors prompt stroke development. Indeed, educating people especially in rural areas should be considered as a priority. Additionally, it should be considered that TV as an important mass media tool plays an important role in educating people. Finally, since the most of the patients stated a relative as their main source of knowledge, educating of even one person within a family could have a crucial role to enhance the public awareness regarding the disease.

## Data Availability

All data generated or analyzed during this study are included in this published article.

## Abbreviations

CPSS: Cincinnati prehospital stroke scale
EMS: Emergency medical services
ICF: Informed Consent Form
t-PA: Tissue plasminogen activator
